# Editorial: Medication safety in COVID-19 management, Volume II

**DOI:** 10.3389/fphar.2023.1175152

**Published:** 2023-03-09

**Authors:** Saibal Das

**Affiliations:** ^1^ Indian Council of Medical Research—Centre for Ageing and Mental Health, Kolkata, India; ^2^ Department of Global Public Health, Karolinska Institutet, Stockholm, Sweden

**Keywords:** COVID-19, medication, pharmacovigilance, safety, vaccine

The COVID-19 pandemic had disrupted the global healthcare ecosystem. To battle this, numerous treatment methods were attempted (WHO, 2022; EMA, 2023; US FDA, 2023). Although a few new medicines were introduced, a majority of the medicines to treat COVID-19 are repurposed ones. Despite lacking adequate safety data, certain medicines were granted emergency use authorizations. Likewise, several COVID-19 vaccines have been developed and widely used globally (CDC, 2023). As the severity of the COVID-19 pandemic has now reduced globally, optimizing medication management in COVID-19 is crucial and it is necessary to discontinue the use of ineffective medicines (Abdool Karim et al., 2022). It is important to conduct a large-scale evaluation of the safety profile of COVID-19 medicines, as well as vaccines (Chiu et al., 2022). This issue has been dedicated to the safety aspects of pharmacotherapy for COVID-19.

Merino et al. aimed to investigate the potential link between the development of transient global amnesia following the administration of COVID-19 vaccines. The World Health Organization VigiBase^®^ database was consulted for all reports of transient global amnesia. Although cerebrovascular, inflammatory, or migrainous mechanisms might underlie this association, the causality could not be ascertained. Significant disproportionality was noticed and the role of several confounding factors could not be nullified.

Jeong et al. conducted a discovery-driven data analysis using the United States Food and Drugs Administration Adverse Event Reporting System database to identify potential drug-drug interactions due to the use of multiple medications in COVID-19 patients and associated adverse events. Remdesivir was discovered to interact with the largest number of concomitant medicines, while hydroxychloroquine was detected to be associated with the most adverse events. The results warrant further large-scale pharmacoepidemiological studies.

Nishimura et al. used an international network of large-scale healthcare databases (electronic health records from Spain and the United States) to generate comprehensive real-world evidence on the role of alpha-1 blockers in preventing COVID-19 complications by minimizing cytokine storm release. Using a meta-analytic approach, the authors demonstrated that there was no evidence of the hypothesized reduction in risks of COVID-19 outcomes from the prevalent use of alpha-1 blockers.

Pineda et al. evaluated the effect of adjunct fluvoxamine treatment along with the standard of care in COVID-19 patients in a prospective observational study in Honduras. A total of 657 COVID-19 outpatients received adjunct fluvoxamine treatment and were monitored for 30 days. The results suggested that adjunct fluvoxamine lowered the relative risk of death, hospitalization, and oxygen requirement in COVID-19 patients.

Amponsah et al. reviewed the current data on the safety of anti-inflammatory medicines (non-steroidal anti-inflammatory drugs and corticosteroids) in COVID-19 management. The review suggests that methylprednisolone, dexamethasone, and ibuprofen could reduce the mortality rate in hospitalized COVID-19 patients. Non-steroidal anti-inflammatory drugs and corticosteroids do not increase the risk of COVID-19 infections and are not associated with adverse outcomes in COVID-19.

Vaccines and medicines to prevent and treat COVID-19 are still evolving. Numerous studies have been completed within a short timeframe (WHO, 2022; EMA, 2023; US FDA, 2023), thereby limiting the detailed safety evaluations of investigational medicines. Hence, it is essential to collect robust short- and long-term safety data for all these medicines. Consideration of the safety concerns in the context of individual and specific stages of the disease is important to formulate a treatment plan. During the pre-clinical development stage, it is necessary to conduct mechanistic studies, develop biomarkers, and perform toxicokinetic modeling of COVID-19 medicines. Likewise, in the vaccine landscape, ensuring an effective and safe vaccination strategy for COVID-19 could be an ideal way to achieve herd immunity (Poland et al., 2020). Hence, it is also important to investigate short- and long-term adverse effects after COVID-19 vaccination. After regulatory approval, a real-world pragmatic approach is required to address the safety aspects of COVID-19 medicines and vaccines. Randomized database studies, chart reviews, claims data, administrative data analyses, computer monitoring, direct care observations, and other pharmacovigilance studies can serve the purpose.
